# G-CSF augments the neuroprotective effect of conditioned medium of dental pulp stem cells against hypoxic neural injury in SH-SY5Y cells 

**DOI:** 10.22038/IJBMS.2021.60217.13344

**Published:** 2021-12

**Authors:** Farahnaz Ahmadi, Zahra Salmasi, Majid Mojarad, Atieh Eslahi, Zahra Tayarani-Najaran

**Affiliations:** 1 Medical Toxicology Research Center, Mashhad University of Medical Sciences, Mashhad, Iran; 2 Nanotechnology Research Center, Pharmaceutical Technology Institute, Mashhad University of Medical Sciences, Mashhad, Iran; 3 Department of Pharmaceutical Nanotechnology, School of Pharmacy, Mashhad University of Medical Sciences, Mashhad, Iran; 4 Department of Medical Genetics, Faculty of Medicine, Mashhad University of Medical Sciences, Mashhad, Iran; 5 Student Research Committee, Mashhad University of Medical Sciences, Mashhad, Iran; 6 Targeted Drug Delivery Research Center, Pharmaceutical Technology Institute, Mashhad University of Medical Sciences, Mashhad, Iran

**Keywords:** Cobaltous chloride, Granulocyte colony-stimulating factor, Hypoxia, Stem cells, Transfection

## Abstract

**Objective(s):**

Dental pulp stem cells (DPSCs) can differentiate into functional neurons and have the potential for cell therapy in neurological diseases. Granulocyte colony-stimulating factor (G-CSF) is a glycoprotein family shown neuroprotective effect in models of nerve damage.

we evaluated the protective effects of G-CSF, conditioned media from DPSCs (DPSCs-CM) and conditioned media from transfected DPSCs with plasmid encoding G-CSF (DPSC-CMT) on SH-SY5Y exposed to CoCl_2_ as a model of hypoxia-induced neural damage.

**Materials and Methods:**

SH-SY5Y exposed to CoCl_2_ were treated with DPSCs-CM, G-CSF, simultaneous combination of DPSCs-CM and G-CSF and finally DPSC-CMT. Cell viability and apoptosis were determined by resazurin (or lactate dehydrogenase (LDH) assay alternatively) and propidium iodide (PI) staining. Western blot analysis was performed to detect changes in apoptotic protein levels. The interleukin-6 and interleukin-10 IL6/IL10 levels were measured with Enzyme-Linked Immunosorbent Assay (ELISA).

**Results:**

DPSCs-CM and G-CSF were able to significantly protect SH-SY5Y against neural cell damage caused by CoCl_2_ according to resazurin and LDH analysis. Also, the percentage of apoptotic cells decreased when SH-SY5Y were treated with DPSCs-CM and G-CSF simultaneously. After transfection of DPSCs with G-CSF plasmid, DPSC-CMT could significantly improve the protection. The amount of β-catenin, cleaved PARP and caspase-3 were significantly decreased and the expression of survivin was considerably increased when hypoxic SH-SY5Y treated with DPSCs-CM plus G-CSF according to Western blot. Decreased level of IL-6/IL-10, which exposed to CoCl_2_, after treatment with DPSCs-CM indicated the suppression of inflammatory mediators.

**Conclusion:**

Combination therapy of G-CSF and DPSCs-CM improved the protective activity.

## Introduction

Migration of MSCs to injured sites and differentiation to mature cells with functional activity is a process of tissue repair. Modulating the immune function is an important effect of MSCs besides the ability to promote cell growth, stimulate proliferation, inhibit cell death and development of pro-angiogenic factors. All modalities via paracrine influence or direct cell to cell contact made them as an appropriate tool to recuperate the injuries to various tissues (1).

HDPSCs (Human dental pulp stem cells) are ecto-mesenchymal derived stem cells, originating from migrating neural crest cells (2). Furthermore, DPSCs express nestin and βIII-tubulin as neuronal lineage markers that support the notion that these cells may be capable to differentiate into neural cells (3). DPSCs also express immunomodulatory factors that stimulate the formation of blood vessels and augment the regeneration and repair of injured nerves. Hence DPSCs are a suitable candidate as a source of replacement cells for injured neuronal cells (4) . 

The mechanism of action after DPSCs transplantation is not fully understood but it is likely to be a paracrine-mediated mechanism, with the secretion of neurotrophic factors coordinated for neuronal survival and axonal regeneration (2).

Treatment with neurogenic factors such as epidermal growth factor (EGF), retinoic acid and fibroblast growth factor (FGF) are effective in the differentiation of DPSCs into neural cells (5).

DPSCs show a higher proliferative rate, higher number of stem/progenitor cells in the population and a greater clonogenic potential compared to BM-MSCs (Bone marrow-mesenchymal stem cells) (6). In addition, they exhibit superior neuroprotective effects in neurological injuries and pathologies as compared with BM-MSCs and ADSCs (Adipose stem cells). This superiority might be related to higher expression of neurotrophic factors including brain-derived neurotrophic factor (BDNF), neurotrophin-3 (NT-3), glial cell-line derived neurotrophic factor (GDNF), nerve growth factor (NGF), platelet-derived neurotrophic factor (PDGF) and vascular endothelial growth factor (VEGF) (2).

DPSCs secretum has been widely used in researches as a cell-free therapeutic tool that decreases the risks of immune reactions and the development of ectopic tissue which are associated with the engraftment of stem cells (7). The DPSCs secretum has high concentrations of fms-related tyrosine kinase 3 (FLT-3), monocyte chemoattractant protein 1 (MCP-1) and granulocyte-macrophage colony-stimulating factor (GM-CSF). 

Compare to BM-MSCs and ADSCs, the DPSCs secretum show higher angiogenic and neurogenic potentials in ectopic transplantation models and exhibit the highest migration capacity. DPSCs secretum also mediate stronger anti-apoptotic effects in a microenvironment challenged by oxidative and serum deprivation (8). 

Due to these properties, DPSCs have been considered as a potential source for cell-based therapy for neural diseases such as Parkinson’s disease (2), Alzheimer’s disease (9), amyotrophic lateral sclerosis (10) and stroke (8).

Granulocyte colony-stimulating factor (G-CSF) is a 19.6-kDa glycoprotein that belongs to the cytokine family of growth factors (11). It is clinically applied for treating neutropenia in humans (12). Several studies have been demonstrated the neuroprotective effect of G-CSF in a variety of experimental brain injury models (13, 14). For example, administration of G-CSF in rats model of focal cerebral ischemia improved the survival rate and neurological behavior, also lowered the infraction volume compared to the vehicle group (15). Neuroprotective effect of G-CSF in a model of Parkinson^’^s disease in male mice demonstrated by reduction of striatal dopamine depletion after MPTP (1-methyl-4-phenyl-1,2,3,6-tetrahydropyridine) application (16).

It was found that the conditioned medium (CM) of DPSCs contains a wide range of bioactive secreted factors (17) so, in this study, we evaluated the neuroprotective effects of simultaneous application of DPSCs-CM and G-CSF on SH-SY5Y cells exposed to CoCl_2_ as an approperiate model of hypoxic neural injury. 

Furthermore, G-CSF gene transfection to DPSCs was used to improve the problem of short half-life of G-CSF, thereby the cellular carrier which provided longer access to G-CSF was designed and prepared and finally the protective effect of conditioned medium derived from transfected cells (DPSC-CM_T_) was investigated. To the best of our knowledge, so far, no combination therapy using DPSC-CM_T_ and G-CSF has been done in any study

## Materials and Methods

***Materials***

Collagenase type I, dispase type Π, fetal bovine serum (FBS), penicillin/streptomycin, trypsin and L-glutamine were purchased from Gibco, USA. Plasmid pAdTrack-CMV was prepared as a gift from Dr. Bert Vogelstein, then green fluorescent protein (GFP) and Human G-CSF genes were inserted in this plasmid and amplified in *Escherichia coli *strain *XL1*-*Blue* and then extracted by a Plasmid Mega Kit (QIAGEN, Germany). Ascorbic acid 2-phosphate, dexamethasone, glycerol 2-phosphate, resazurin and alpha modification of Eagle’s medium (α- MEM) were obtained from Sigma-Aldrich. Oil Red O and Alizarin Red S were taken from Santa Cruz Biotechnology (Santa Cruz, CA, USA). Monoclonal antibodies were taken from Cell Signaling, USA. IL-6 (CN: KPG-HI6P) and IL-10 (CN: KPG-HI10P) ELISA kits were purchased from Karmania Pars Gene. Lipofectamine 3000 reagent was obtained from Thermofisher USA Invitrogen.

***Cell culture and treatments***

*Isolation and expansion of* *dental pulp stem cells*

Pulps of third molar teeth were obtained from 18-29 years adults due to orthodontic surgery at the Dental Clinic of the Institute of Dental and Craniofacial of Mashhad University of Medical Sciences based on institution ethical approval. Isolation of dental pulp stem cells was carried out using the protocole previously described (18). Brifely, dental pulps were cut into 2-3 small fragments and enzymatically digested using 3 mg/ml of collagenase type I and 4 mg/ml of dispase type Π (Gibco, USA) solution for 45 minutes at 37°C. Following centrifugation at 500 g for 5 min, the cell pellet was suspended and seeded in a culture flask with alpha modification of Eagle’s medium (α- MEM; Sigma, Germany) supplemented with 20% fetal bovine serum (FBS; Gibco, USA) and the final concentration of 1% penicillin/streptomycin (Gibco, USA) and 0.01% amphotericin B (Simintak, Iran). The culture flask was cultivated at 37°C in 5% CO_2. _The medium was changed every three days until stem cells reached 70% confluence (19). No specific blinding or randomization was used in the current study. 

*Hypoxia induction in SH-SY5Y cells by treating with CoCl*_2_

SH-SY5Y cells were cultured in α-MEM medium supplemented with 10% fetal bovine serum, 1% penicillin/streptomycin with the density of 1×10^4 ^cells/well at 37°C and 5% CO_2 _humidified atmosphere. Hypoxia was induced in SH-SY5Y cells by treating with CoCl_2_ at the concentration of 0.6 and 1.2 mM and different times of exposure (6, 12, 24 and 48 hr) to determine the optimum treating situation for subsequent experiments (20). The cell viability was assessed by resazurin (7-Hydroxy-3*H*-phenoxazin-3-one 10-oxide) assay. In brief, 20 µl of resazurin (0.15 mg/ml) (Sigma, Germany) was added to each well and incubated at 37 °C for 4 h. The absorbance was measured at 600 nm via Synergy H4 Multi-Mode microplate reader (BioTek, Winooski, USA). 

*Flow cytometry analysis for surface markers*

DPSCs at passage 3 were used to determine the human surface markers associated with mesenchymal and hematopoietic lineages using the following monoclonal antibodies (mAbs); mouse anti human CD45 FITC, mouse anti human CD34 FITC, mouse anti human CD29 FITC and mouse anti human CD44 FITC. Flow cytometry was performed using BD Calibur flow cytometer (Germany) and data were analyzed by FlowJo version 7.6 software (Flowjo LLC, USA).

*Preparation of DPSCs conditioned medium*

To prepare the conditioned medium, DPSCs at 3-5^th^ passages with the density of 1×10^6^ cells/plate were incubated in serum-free α-MEM for 48 h in 37ºC and 5% CO_2_. Then the supernatant was centrifuged at 500 g at 4°C to remove detached cells. The conditioned medium was kept at -80 °C until use (21).

*Protective effects of DPSCs-CM and G-CSF on SH-SY5Y cells in hypoxic condition*

SH-SY5Y cells at the density of 1×10^4 ^cells/well were incubated with CoCl_2_ (0.6 and 1.2 mM). DPSCs-CM and DPSC-CM plus G-SCF at concentration of 10, 100 and 500 ng/ml were added to SH-SY5Y cells and incubated at 37°C for 6, 12, 24 and 48 h. Assessment of cell viability was performed by resazurin (Sigma, Germany) analysis.

*IL-6 and IL-10 release assessment*

IL-6 and IL-10 protein levels in culture supernatants were measured using commercial ELISA kits (Karmania Pars Gene). Standard solutions were prepared according to manufacture protocol and 50 µL of each standard and supernatants from DPSCs were added to wells coated with IL-6 or IL-10 separately. Plates were shacked gently at 200 rpm for 2 h at 37 °C. After that, 50 µL of detection antibody were added to each well and incubated at 200 rpm for 1 h at 37°C. Plates were washed 3 times and incubated with Avidin-HRP at 200 rpm for 30 min at room temperature. Finally, plates were washed extensively and incubated with substrate solution for 15 min at room temperature, followed by the addition of 25 µL of stop solution. The absorbance was measured at 450 nm via Synergy H4 Multi-Mode microplate reader (BioTek, Winooski, USA). 

*Western blot technique*

SH-SY5Y cells were plated in a 12-well plate at a density of 1×10^6^ cells/well and incubated for 24 h at 37 °C and then treated according to section 2-4. Cells lysates were extracted by adding cell lysis buffer. An equal amount of protein extracts (50 µg) were subjected to 12% SDS-PAGE and subsequently transferred to polyvinylidene difluoride membranes (Bio-Rad, USA). Membranes were blocked with 5% skim milk in TBST (Buffered Saline Tween 20) at room temperature for 2 h and probed with primary antibodies (Cell Signaling, USA) against survivin, cleaved PARP, β-catenin and cleaved caspase-3 at 1:1000 dilution overnight at 4°C. β-actin at a 1:1000 dilution wasused as the vloading control. Following three times washing with TBST, blots were incubated with rabbit anti-rabbit horseradish peroxidase-conjugated secondary antibody (1:2000). Bands were viewed using ECL (Enhanced Chemiluminescent) detection system. Results were normalized, using β-actin as the reference.

*Transfection of plasmid encoding G-CSF to DPSCs*

DPSCs were plated in 24-well plates, cultured with α-MEM supplemented with 10% FBS and 1% penicillin/streptomycin, and maintained overnight at 37°C, 5% CO_2_ in a humidified atmosphere before transfection. The protocol was performed according to the supplier’s instructions. Briefly, Lipofectamine 3000 reagent (Thermofisher USA Invitrogen), was diluted in Opti-MEM^TM^. Polyplexes were prepared by adding a calculated amount of Lipofectamine 3000 solution in Opti-MEM to equal volumes of plasmid DNA solution (1.5 μg per well plasmid encoding G-CSF-GFP) with gentle pipetting and incubated for 30 min at room temperature. After replacing the cell culture medium with serum-free MEM α, the transfection mixture was added to the cells and incubated for 4 h at 37°C. Then, the medium was replaced with serum supplemented MEM α and the cells were incubated at 37ºC for 48 h. After that, wells were analyzed for transfected cells under a fluorescent microscope (22).

*Assessment of LDH release from SH-SY5Y cells in hypoxic condition*

LDH release assay was performed to analyze the cellular damage caused by a hypoxic condition in SH-SY5Y cells exposed to CoCl_2_. Briefly, 1×10^5^ cells were seeded per well of 12 well plates. After 24 h, DPSC-CM, DPSC-CM_T_ and DPSC-CM plus G-CSF (10, 500 ng/ml) were added to each well, then incubated with CoCl_2_ as described above. The cell supernatants were collected in separate tubes, then mixed with LDH reaction reagent for 30 min at room temperature. The absorbance was measured at 490 nm via Synergy H4 Multi-Mode microplate reader (BioTek, Winooski, USA). 

*Flow cytometry analysis for the cell apoptosis*

1×10^5 ^SH-SY5Y cells/well were treated with DPSC-CM, DPSC-CM_T_ and DPSC-CM plus G-CSF (10, 500 ng/ml) and exposed to 0.6 mM of CoCl_2_ for 24 h. Then cells were harvested and washed twice with phosphate-buffered saline (PBS) and centrifuged at 500 g for 5 min. 300 μL of the PI solution was added to each tube and incubated for 30 min at 37^o^C and finally analysed with a flow cytometer. 

***Statistical analysis***

The data was reported as the mean ± SD of three independent experiments each in triplicate. The data were analyzed using Graph Pad Prism software, version 6.0. 

Cell viability was analyzed using two-way analysis of variance (ANOVA) followed by Tukey as post-test. One-way ANOVA and Tukey’s multiple comparisons test was used to assess the significant differences between the experimental groups in other tests. *P* value <0.05 was considered to indicate a statistically significant difference.

***Ethics approval***

The protocol for this work (No. 970850 and 960882) and reference number (IR.MUMS.sp.REC.1396.202_October14,2020) have been approved by Mashhad University of Medical Sciences.

## Results

***Hypoxia induction and investigation of the protective effect of DPSCs-CM and DPSCs-CM plus G-CSF***

**Figure 1:**
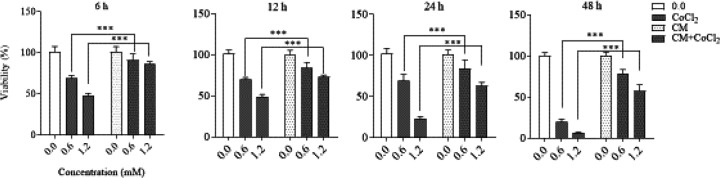
Effect of CoCl_2_ on SH-SY5Y cells viability and DPSCs-CM on SH-SY5Y exposed to CoCl_2_. 1×10^4^ cells/well of SH-SY5Y cells were exposed to 0.6 and 1.2 mM conncentration of CoCl_2_ for 6, 12, 24 and 48 hr. The cell viability was assesed by resazurin asssay. The value presented as mean ± SD (n=9). ***P<0.001

CoCl_2 _at the concentration of 0.6 and 1.2 mM significantly (*P*˂ 0.001) reduced the cell viability of SH-SY5Y after 6, 12, 24 and 48 h exposure [F (3, 48) = 1422] (Figure 1). A significant enhancement in the viability of cells exposed to hypoxia was seen when cells were treated with DPSCs-CM (*P*< 0.001) (Figure 1).

**Figure 2:**
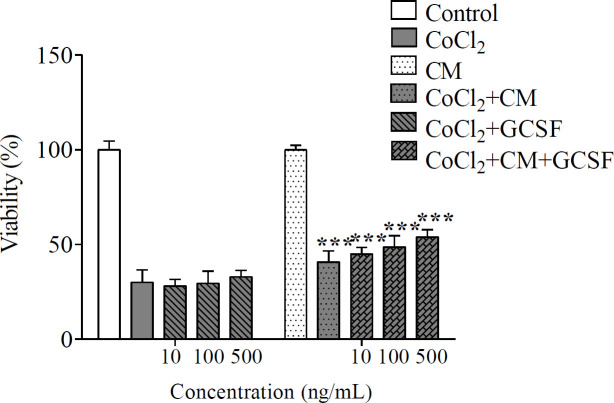
Protective effect of G-CSF and DPSCs-CM on SH-SY5Y exposed to CoCl_2_. 1×10^4^ cells/well were exposed to hypoxic condition by 0.6 mM of CoCl_2_ for 24 hr. Cells were treated with DPSCs-CM and different concentration of G-CSF (10, 100, 500 ng/ml) for 24 hr Value presented as mean±SD (n=9), ***P<0.001 in comparison with CoCl_2_

G-CSF: Granulocyte macrophage-colony stimulating factor; DPSCs-CM: Dental pulp stem cells- conditioned medium

In the next step, the results of the viability assay confirmed the neuroprotective effect of DPSCs-CM and DPSCs-CM plus G-CSF on SH-SY5Y cells against the hypoxic condition. DPSCs-CM plus G-CSF (10, 100 and 500 ng/ml) significantly reduced the cellular damage in SH-SY5Y cells exposed to CoCl_2 _[F (4, 10) = 118.7, *P*<0.001] (0.6 mM at 24 hr) determined by viability assay (*P*=0.0244, 0.0030 and 0.0002 respectively) (Figure 2). DPSCs-CM plus G-CSF had superior protective activity in comparison with G-CSF or DPSCs-CM alone [F (4, 10) = 88.07, *P*<0.001] (*P* values for GCSF10, 100 and 500 vs. CM:GCSF10, 100 and 500 were 0.0083; 0.0024 and <0.001, respectively)

***Assessment of IL-6 and IL-10 release***

**Figure 3:**
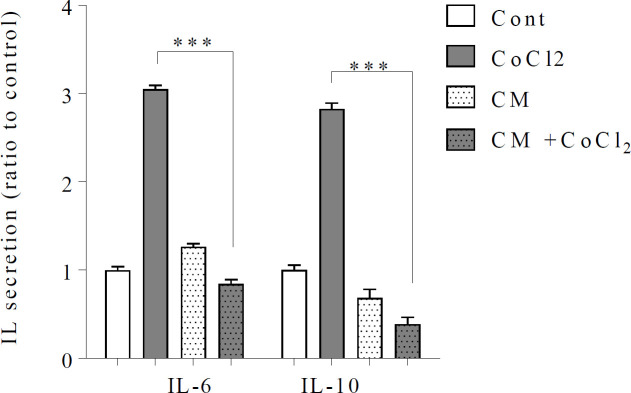
Effect of CoCl_2_ on secretion of IL-6 and IL-10 in SH-SY5Y cells. The value presented as mean ± SD (n=9), ***P<0.001 in comparison with CoCl_2_

Levels of Interleukin 6 and 10 were evaluated as important factors involved in neural protection. The results of ELISA test showed that the levels of IL-6 [F (3, 8) = 1182, *P*<0.001] and IL-10 [F (3, 8) = 637.8, *P*<0.001] in cells exposed to 0.6 mM of CoCl_2 _for 24 hr were increased compared to control and significantly decreased when cells were treated with DPSCs-CM compared to the hypoxia group (*P*˂0.001) (Figure 3).

***Analysis of survivin, cleaved PARP, β-catenin and cleaved caspase-3 proteins by western blot***

**Figure 4:**
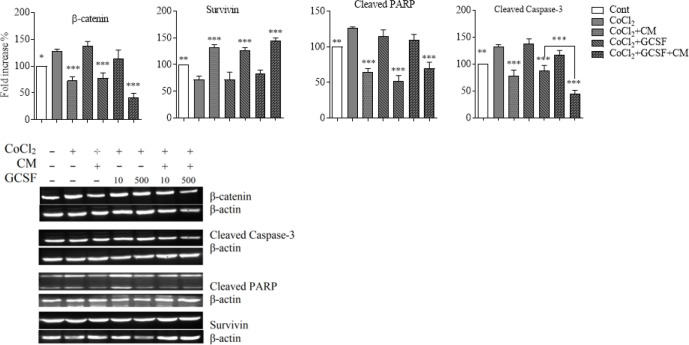
Western blot analysis of SH-SY5Y cells to detect the effect of DPSCs-CM and G-CSF on expression of β-catenin, cleaved caspase-3, cleaved PARP, and survivin on hypoxic condition. 1×10^5^ cells/well of SH-SY5Y were exposed to 0.6 mM of CoCl_2_ then treated with DPSCs-CM and G-CSF (500 ng /ml) for 24 hr and the expression of Survivin, Cleaved PARP], β-catenin, and Cleaved Caspase-3 was determined by western blot. Value presented as mean ± SD (n=9), **P<0.01, ***P<0.001

PARP: Poly (ADP-ribose) polymerase; G-CSF: Granulocyte macrophage-colony stimulating factor; DPSCs-CM: Dental pulp stem cells- conditioned medium

Western blotting was performed to detect changes in apoptotic protein levels such as β-catenin, cleaved PARP, cleaved caspase-3 and survivin. As shown in Figure 4, the expression of survivin significantly increased in cells treated with DPSCs-CM, G-CSF 500 and G-CSF 500 plus DPSCs-CM [F (4, 10) = 99.13, *P*<0.001] (*P* ˂0.001) compared to cells treated with 0.6 mM of CoCl_2_. Whereas the amount of β-catenin [F (4, 10) = 64.82, *P*<0.001], cleaved caspase-3_[F (4, 10) = 97.36, *P*<0.001], and cleaved PARP [F (4, 10) = 79.30, *P*<0.001] were significantly decreased in cells treated with DPSCs-CM, G-CSF 500 and DPSCs-CM plus G-SCF 500 (*P*˂0.001) compared to cells treated with 0.6 mM of CoCl_2_. DPSCs-CM plus G-CSF 500 had superior protective activity in comparison with G-CSF 500 alone for β-catenin (*P*=0.0027) and caspase 3 (*P*<0.001).

***Transfection experiment and evaluation of LDH release from SH-SY5Y*** ***cells ***

**Figure 5:**
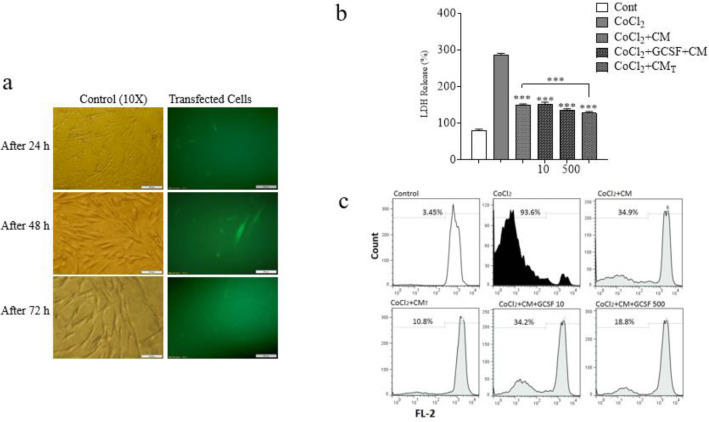
a) Investigation of G-CSF-GFP expression in DPSCs transfected with lipofectamin using fluorescent microscopy. All scale bars represent 100 μm. b) LDH release and c) flow cytometery analysis of SH-SY5Y cells treated with DPSCs-CMT and G-CSF in hypoxic condition. 1×10^5^ cells/well of SH-SY5Y were treated with 0.6 mM of CoCl2, DPSCs-CM and G-CSF (10 and 500 ng /ml) for 24 hr and the amount of LDH release was determined. Value presented as mean ± SD (n=9), ***P< 0.001 in comparison with CoCl_2_

To design a cellular carrier which could express G-CSF in addition to conditioned media, we transferred the plasmid encoding G-CSF-GFP to DPSCs. The expression of this plasmid was investigated using fluorescent microscopy. As indicated in Figure 5a, 48 hr after transfection, gene expression increased in DPSCs cells.

Afterwards, results of the LDH assay from SH-SY5Y cells exposed to CoCl_2_ confirmed the neuroprotective effect of DPSCs-CM, DPSCs-CM_T_ and DPSCs-CM plus G-CSF on SH-SY5Y cells against the hypoxic condition. DPSCs-CM, DPSCs-CM_T_ and DPSCs-CM plus G-CSF significantly reduced the cellular damage in SH-SY5Y cells exposed to CoCl_2 _(0.6 mM at 24 hr) determined by LDH assay [F (5, 12) = 755.5, *P*<0.001] (Figure 5b). DPSCs-CM_T_ had superior protective activity in comparison with DPSCs-CM (*P*˂0.001). 

***Anti-apoptotic effect of DPSCs-CM***_T_ ***and G-CSF on SH-SY5Y cells exposed to CoCl***_2_ ***by Flow cytometry method***

**Figure 6:**
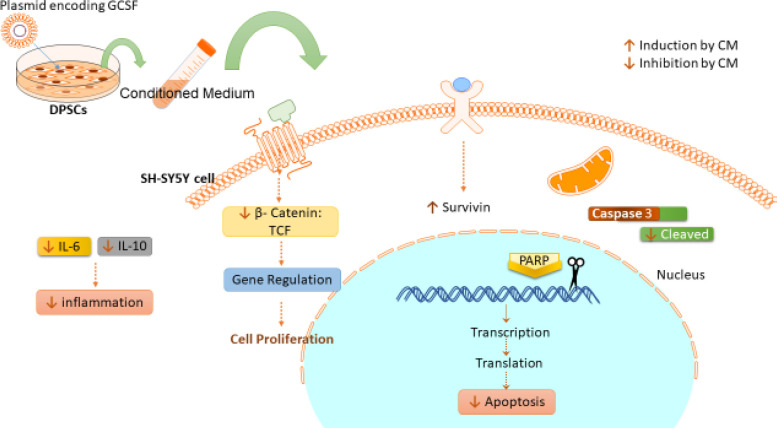
Schematic representation of protective mechanism of DPSCs-CMT and G-CSF in SH-SY5Y cells exposed to hypoxic condition

DPSCs-CM: Dental pulp stem cells- conditioned medium; G-CSF: Granulocyte macrophage-colony stimulating factor

To investigate the protective effect against apoptosis caused by CoCl_2_, propidium iodide was used. Flow cytometry analysis of SH-SY5Y cells stained with PI revealed that exposure to CoCl_2_ (0.6 mM) for 24 hr increased apoptotic cells as compared to control (Figure 5c, Figure 6). DPSCs-CM, DPSCs-CM_T_ and DPSCs-CM plus G-CSF (500 ng/ml) significantly decreased the apoptotic cells in SH-SY5Y cells exposed to CoCl_2 _(*P*˂0.001) compared to hypoxic condition. DPSCs-CM_T_ had superior protective activity in comparison with DPSCs-CM or DPSCs-CM plus G-CSF. 

## Discussion

In the present study, we showed that combination therapy of G-CSF and DPSCs-CM could significantly improve the protective activity against neural cell damage caused by CoCl_2_. After that, the cellular carrier was designed with transfection of plasmid encoding G-CSF to provide conditioned media containing G-CSF. The results showed that DPSC-CM_T _caused considerable protection against hypoxia.

Cell therapy using MSCs has emerged as a novel and promising neuroprotective strategy in neural diseases such as ischemic stroke, Alzheimer and Parkinson’s diseases. Accumulating data have suggested DPSCs as an important candidate for cell therapy. DPSCs may enhance neuronal survival and neurite outgrowth via a paracrine mechanism. In addition, evidence indicated that DPSCs play an important role in neurogenesis, neural maintenance and repair due to the secretion of several neurotrophic factors (23). Studies have been shown that DPSCs can promote neuroprotection after nerve injury (2, 24). 

In a study, the secretum obtained from DPSCs was able to reduce the cytotoxicity and apoptosis caused by the amyloid-beta peptide in Alzheimer’s disease (9). Administration of DPSCs during acute ischemic stroke provided neuroprotective effects by modulating inflammation and blood-brain barrier permeability in post-ischemia/reperfusion brain injury in rats (25). Intracerebral transplantation of DPSCs into ischemic regions of the brain significantly improves the forelimb sensory-motor function in a rodent model after 4 weeks post-treatment (26).

SH-SY5Y is a thrice cloned subline of SK-N-SH cells which were originally obtained from a bone marrow biopsy of a neuroblastoma patient in the early 1970s (27). In this study, the cytotoxicity of CoCl_2_ on SH-SY5Y cells was demonstrated at the concentration of 0.6 and 1.2 mM at 6, 12, 24 and 48 hr (20). The culture of SH-SY5Y cells with DPSCs-CM indicated the neuroprotective effect of DPSCs-CM by increasing the cell viability. Similar to our study, the therapeutic potency of MSCs-CM has been explored in an experimental model of spinal cord injury (28), brain injury (29), bone defects (30), and ischemic heart disease (31).

Several studies have been reported that hematopoietic cytokines like granulocyte macrophage-colony stimulating factor (GM-CSF), G-CSF, or erythropoietin had neuroprotective effects and has an important role in recovering the neurologic functions after central nervous system injury (32). 

G-CSF is a hematopoietic growth factor that plays important role in hematopoiesis (33). Number of different cell types such as endothelium, macrophages and some immune cells produce G-CSF and stimulate the bone marrow to release stem cells and granulocytes. 

The receptor of G-CSF is expressed on precursor cells in the bone marrow which when activated, initiates proliferation and differentiation into mature granulocytes. It has also been shown that neurons in the brain and spinal cord express G-CSF receptor and their activation by G-CSF induce neurogenesis, increases neuroplasticity and reduces apoptosis (34). Moreover, activation of G-CSF receptor resulted in stimulating survival, proliferation and development of neuronal stem cells. Usage of G-CSF as a therapy for ischemic stroke was conducted in a phase I/II clinical trial. Patients who received G-CSF showed greater improvement in neurologic functioning between baseline and 12-month follow-up than in the control group (35).

Multiple studies have indicated the neuroprotective effect of G-CSF in a variety of *in vivo* brain injury models (36). It was reported that treatment with G-CSF promotes somatic growth, prevented brain atrophy and improved long-term neurological outcome in the neonatal hypoxic-ischemia model (37). G-CSF administration after middle cerebral artery occlusion revealed a significant reduction in the amount of edematous tissue, brain water content and cortical lesion volume (38).

Co-administration of G-CSF with BM-MSCs produced synergistic beneficial effects by promoting cell proliferation and differentiation of bone marrow stem cells led to early neuronal development, reduction the cerebral infarct size and improved the brain regeneration and functional recovery in an experimental mouse model of cerebral ischemia (39). 

The effect of combinational therapy of G-CSF and BM-MSCs was evaluated in Sprague-Dawley rats after stroke (40). Although, the combination therapy produced remarkable neurogenesis in the formerly infarct core and beyond in the islet of regeneration, it wasn’t more efficient in reducing the mortality rate and improving the post-stroke recovery than G-CSF treatment alone (40). Adjunct treatment of G-CSF with hUCB-MSC (human umbilical cord blood-derived mesenchymal stem cells) in traumatic brain injury (TBI) in adult rats indicated synergetic effect in functional improvement in TBI rats than that exerted by monotherapy with hUCB or G-CSF (41).

In this study, we transfected DPSCs with plasmid encoding G-CSF-GFP. It was shown that 48 h after transfection, the expression of G-CSF increased in DPSCs. Moreover, DPSCs-CM_T_ improved the protective effect which caused a decrease in apoptotic cells and cellular damage induced by CoCl_2_. Western blotting was used to detect changes in apoptotic protein levels such as β-catenin, cleaved PARP, cleaved caspase-3 and survivin.

The Wnt signaling pathway has a role in regulating diverse cellular processes, including cell proliferation and cell death. Following the activation of Wnt/β-catenin pathway, cytoplasmic β-catenin becomes stabilized, enters the nucleus and interacts with transcription factors, notably TCF/LEF to regulate the transcription of target genes (42). 

Wnt/β-catenin pathway activation contributes to functional recovery and induces neuroprotective processes and neurogenesis after focal cerebral ischemia (43). Also, it was shown that the inactivation of β-catenin by small interfering RNA increased the ischemia-induced infarct volume in rats (44). 

Hypoxia may induce the activation of the Wnt signaling pathway in the adult brain. Dysfunction of the Wnt/β-catenin signaling pathway has been linked to neurodegenerative disorders such as schizophrenia, autism and Alzheimer’s disease (45, 46). Overexpression of β-catenin in NIH-3T3 cells and human H1299 cells mimics the induction by apoptotic stimuli of transcriptionally active p53 (47). Destabilization of β-catenin through missense mutation of presenilin-1, which is the most commonly mutated gene in familial Alzheimer patients resulted in inducing neuronal apoptosis (48). 

The wnt signaling pathway regulates apoptosis through a variety of mechanisms including those through SFRP2 (secreted Frizzled-related protein-2) gene expression, wnt-BMP signaling, GSK 3-β-NF-κBeta, β-catenin, c-Jun N-terminal kinase signaling, or gene expression of Dickkopf-1, nemo, sox 10 and tau (49).

PARP-1 (poly (ADP-ribose) polymerase-1) is a nuclear mediator that performs central roles in the repair of damaged DNA, In addition, PARP-1 plays important roles in vasoconstriction, transcription, cardiac remodeling, regulation of astrocyte and microglial function, aging and long term memory (50). Activation of caspase-mediated cell death through the cleavage and activating effector which drive the process of apoptosis. Cleavage of PARP-1 by caspases is considered to be a hallmark of apoptosis and has been implicated in several neurological diseases like Alzheimer’s disease, cerebral ischemia, multiple sclerosis, traumatic brain injury, Parkinson’s disease, NMDA-mediated cytotoxicity and brain tumors (51).

Survivin is a unique member of the inhibitor of the apoptosis gene family. The consequence of the interaction of caspases-3 with survivin is the inhibition of apoptosis (52).

A decrease in the amount of β-catenin also inhibiting the cleavage of caspase-3 and PARP established the neuroprotective effect of DPSCs-CM and G-CSF on SH-SY5Y cells exposed to the hypoxic condition. On the other side, increased levels of survivin, imply the positive effect of DPSCs-CM and G-CSF on neuroprotection and inhibition of apoptosis.

IL-6 is a pleiotropic and multifunctional cytokine that plays an important role in cell proliferation, differentiation, survival and apoptosis (53). IL-6 maintains the homeostasis in the brain by directing neurogenesis, astrogliosis, microgliosis and controlling blood-brain barrier integrity (54). The role of IL-6 in the injured brain is controversial. In one study, it was reported that an increased level of IL-6 may lead to exacerbation of cerebral ischemic damage by increasing harmful mediators and mediating inflammatory cascades (55). On the other hand, Swartz *et al*., reported that IL-6 promotes post-traumatic healing in the nervous system by increasing angiogenesis (56). 

The neuroprotective effect of MSCs in hypoxic-ischemic brain damage (HIBD) rats was demonstrated to be mediated by endogenous IL-6. It performed its anti-apoptotic role via the IL-6/STAT3 signaling pathway (57). 

The level of IL-6 in our study was decreased after subjecting hypoxic SH-SY5Y cells to DPSCs-CM compared to cells exposed to CoCl_2_ which support the neuroprotective effects of DPSCs-CM.

Interleukin-10 (IL-10) is a potent anti-inflammatory cytokine and plays a critical role in balancing immune responses in order to entangle chronic inflammatory diseases (58). The level of IL-10 was increased in the hypoxic condition in our study. However, treatment of cells exposed to hypoxic conditions with DPSCs-CM resulted in to decrease in the secretion of IL-10 compared to the control group. It is well documented that the levels of IL-10 significantly augmented in the serum and CSF after traumatic brain injury (TBI) (59). In experimental models, it was evidenced that IL-10 is implicated in neuroprotective activity in TBI (60). Conversely, in other studies, it was speculated that elevated IL-10 levels correlate with severity and mortality in severe TBI. Furthermore, it was reported that higher levels of IL-10 in CSF have significantly increased mortality both in pediatric and in adult patients (60).

According to these studies, it was found that the role of IL-10 is changeable in different conditions of neurodegenerative diseases.

Overall, we can conclude that combination therapy of G-CSF and DPSCs-CM can improve the neuroprotective activity of DPSCs on neural cells exposed to hypoxic condition. These results were confirmed by evaluating the apoptotic cells which indicated the anti-apoptotic effect of DPSCs-CM, DPSCs-CM_T_ and DPSCs-CM plus G-CSF with the superior effect of DPSCs-CM_T_ on SH-SY5Y cells exposed to CoCl_2_. It seems that DPSCs-CM and G-CSF were able to regulate the apoptosis pathways which resulted in to decrease in the β-catenin and cleaved form of caspase-3 and PARP-1 also increase in the level of survivin (Figure 6). Furthermore, the results of the LDH assay emphasize the neuroprotective effect of DPSCs-CM, DPSCs-CM_T_ and G-CSF. 

It is believed that DPSCs-CM_T_ and DPSCs-CM plus G-CSF had a superior protective effect in comparison with DPSCs-CM or G-CSF alone to fight against the hypoxic condition. With transfection of plasmid encoding G-CSF, we designed a cellular carrier which has two advantages: 1- These engineered_cells could express G-CSF for a longer time and may overcome the short half-life of G-CSF. 2- This cellular carrier, probably, play the role of combination therapy itself by secretion of G-CSF in addition to conditioned media. Further clinical studies may elucidate the preference of engineered-cell therapy in neural injuries.

DPSCs-CM: Dental pulp stem cells- conditioned medium

## Conclusion

Overall, we can conclude that combination therapy of G-CSF and DPSCs-CM can improve the neuroprotective activity of DPSCs on neural cells exposed to hypoxic conditions. These results were confirmed by evaluating the apoptotic cells which indicated the anti-apoptotic effect of DPSCs-CM, DPSCs-CM_T_ and DPSCs-CM plus G-CSF with the superior effect of DPSCs-CM_T_ on SH-SY5Y cells exposed to CoCl_2_. It seems that DPSCs-CM and G-CSF were able to regulate the apoptosis pathways which resulted in to decrease in the β-catenin and cleaved form of caspase-3 and PARP-1 also increase in the level of survivin. Furthermore, the results of the LDH assay emphasize the neuroprotective effect of DPSCs-CM, DPSCs-CM_T_ and G-CSF. 

It is shown that DPSCs-CM_T_ and DPSCs-CM plus G-CSF had a superior protective effect in comparison with DPSCs-CM or G-CSF alone to fight against the hypoxic condition. With transfection of plasmid encoding G-CSF, we designed a cellular carrier which has two advantages: 1- These engineered cells can express G-CSF for a longer time and may overcome the short half-life of G-CSF. 2- This cellular carrier, probably, play the role of combination therapy itself by secretion of G-CSF in addition to conditioned media. Further clinical studies may elucidate the preference of engineered-cell therapy in neural injuries.

## Authors’ Contributions

FA performed the experiments, computations, analyzed the data, and wrote the manuscript. ZS conceived, designed, and supervised the project and approved the final manuscript. MM designed and supervised some parts of the project. AE performed the experiments, computations. ZT conceived, designed, and supervised the project, wrote the manuscript, provided financial support and approved the final draft of the manuscript.

## Data Availability

The datasets generated during and/or analyzed during the current study are available from the corresponding author on reasonable request.

## Conflicts of Interest

The authors declare no conflict of interest, financial or otherwise.
